# Radiographs Reveal Exceptional Forelimb Strength in the Sabertooth Cat, *Smilodon fatalis*

**DOI:** 10.1371/journal.pone.0011412

**Published:** 2010-07-02

**Authors:** Julie A. Meachen-Samuels, Blaire Van Valkenburgh

**Affiliations:** Department of Ecology and Evolutionary Biology, University of California Los Angeles, Los Angeles, California, United States of America; Raymond M. Alf Museum of Paleontology, United States of America

## Abstract

**Background:**

The sabertooth cat, *Smilodon fatalis*, was an enigmatic predator without a true living analog. Their elongate canine teeth were more vulnerable to fracture than those of modern felids, making it imperative for them to immobilize prey with their forelimbs when making a kill. As a result, their need for heavily muscled forelimbs likely exceeded that of modern felids and thus should be reflected in their skeletons. Previous studies on forelimb bones of *S. fatalis* found them to be relatively robust but did not quantify their ability to withstand loading.

**Methodology/Principal Findings:**

Using radiographs of the sabertooth cat, *Smilodon fatalis*, 28 extant felid species, and the larger, extinct American lion *Panthera atrox*, we measured cross-sectional properties of the humerus and femur to provide the first estimates of limb bone strength in bending and torsion. We found that the humeri of *Smilodon* were reinforced by cortical thickening to a greater degree than those observed in any living felid, or the much larger *P. atrox*. The femur of *Smilodon* also was thickened but not beyond the normal variation found in any other felid measured.

**Conclusions/Significance:**

Based on the cross-sectional properties of its humerus, we interpret that *Smilodon* was a powerful predator that differed from extant felids in its greater ability to subdue prey using the forelimbs. This enhanced forelimb strength was part of an adaptive complex driven by the need to minimize the struggles of prey in order to protect the elongate canines from fracture and position the bite for a quick kill.

## Introduction

Few extinct predators are as well-known as the saber tooth cats, which are touted for their prowess as ultimate mammalian predators [1], [2]. Numerous studies of the skull, teeth, and neck of sabertooth cats have examined how they may have dispatched their prey, e.g. [1], [3]–[10]. A consensus has emerged that the sabertooth cat *Smilodon fatalis* probably differed from modern big cats in making relatively quick kills using directed slashing bites to the throat rather than a suffocating bite, as is typical of extant big cats such as lions. In association with this, *Smilodon* had robust forelimbs that were instrumental in restraining prey so that the killing bite or bites could be made with minimal risk of breaking the elongate canine teeth [11]–[13]. From external measurements of the forelimb bones, it appears that they were relatively thick for their length [2], [12], [13] and therefore probably more resistant to bending and compressive loads; however, more accurate estimates of strength require data on both external diameters and cortical bone thickness.

Radiographs allow the measurement, in any plane of interest, of both endosteal and subperiosteal bone diameters and also cortical area and thickness. These measures can be used to estimate bone strength in axial compression (cortical area) as well as to calculate moments of area that reflect resistance to bending and torsion. Previous workers have used cross-sectional properties of mammalian limb bones in various species to identify differences in the pattern of forelimb versus hind limb use, e.g. [14], [15], to estimate body mass in extant and extinct taxa, e.g. [16]–[18], to document significant declines in human bone strength over time despite relatively constant external bone dimensions [19], and even to document asymmetries in left vs. right arm strength in modern human athletes [20].

Despite the many uses of cross-sectional properties in the literature, there is substantial debate about the straightforwardness of these measurements. Studies [21]–[23] warn that cross-sections of limb midshafts do not always indicate repeated loading patterns in all animals in the same way and cross-sectional geometry of long bones does not correlate well with strain patterns. These authors recommend that *in vivo* data be used whenever possible to get accurate assessments of strain patterns and bone loading. With these caveats, there is still evidence that strain does play a role in bone remodeling, however, this role is more complex than originally thought [24]–[26]. When *in vivo* studies are not possible, as in fossil species, variations in bone structure can still be effective indicators of locomotor modes and limb use among closely related species [27]. Comparisons of bone cross-sectional properties can also be good estimators of mechanical ability, if the comparisons are kept to closely related groups that share similar body plans and locomotor ecologies, such as living and extinct felids [27].

Quadruped limbs are used for weight-bearing as well as other activities, such as climbing, digging, swimming and grappling with prey. In the case of large cats, the hind limb functions primarily in weight-bearing and propulsion, whereas the forelimb functions in weight-bearing, climbing, and prey killing [28], [29]. Of course, the hind limbs contribute during climbing but their role is still largely propulsive whereas the forelimbs both grasp the trunk and pull the body upwards. Thus, it might be expected that the humeri of cats that are arboreal or take prey larger than themselves would exhibit greater cortical thickening than expected based on body mass alone. Surprisingly, this does not appear to be the case, as a recent study found that humeral cross-sectional properties were better predictors of body mass than prey size or locomotor habits in extant felids [30]. Given the proposed greater need for strong forelimbs, we hypothesized that the humerus of *Smilodon* would exhibit significantly greater resistance to bending and compression relative to other cats, whereas its femur would scale as expected for its body size.

Here we provide the first quantitative analysis of the ability to resist bending stresses in the forelimbs of *S. fatalis* using radiographic images, and compare it to living cats; and because *Smilodon* was as large, or larger than the largest extant felids, we also included the extinct American lion (*Panthera atrox*) in our sample as a much larger species with forelimb morphology that is similar to its extant sister group, *Panthera leo* [31], and unlike *Smilodon*.

## Results

**Figure 1:**
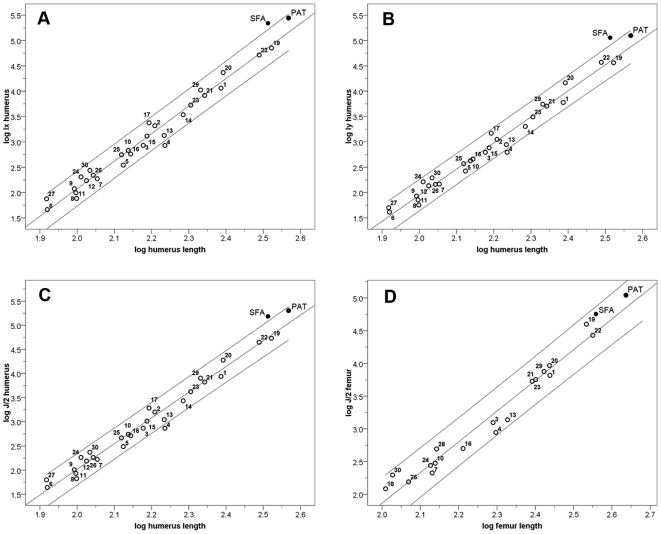
Regressions of humerus and femur cortical measurements against lengths. Log_10_/log_10_ regressions of a) humerus I_x_; b) humerus I_y_; c) humerus J/2 against humerus length; and d) femur J/2 against femur length. *S. fatalis* was not included in regression calculations. Confidence intervals (95%) were based on individual species. Regression statistics are in Table 1. PAT = *Panthera atrox*, SFA = *Smilodon fatalis*, see Table S1 for extant species numbers.

**Figure 2:**
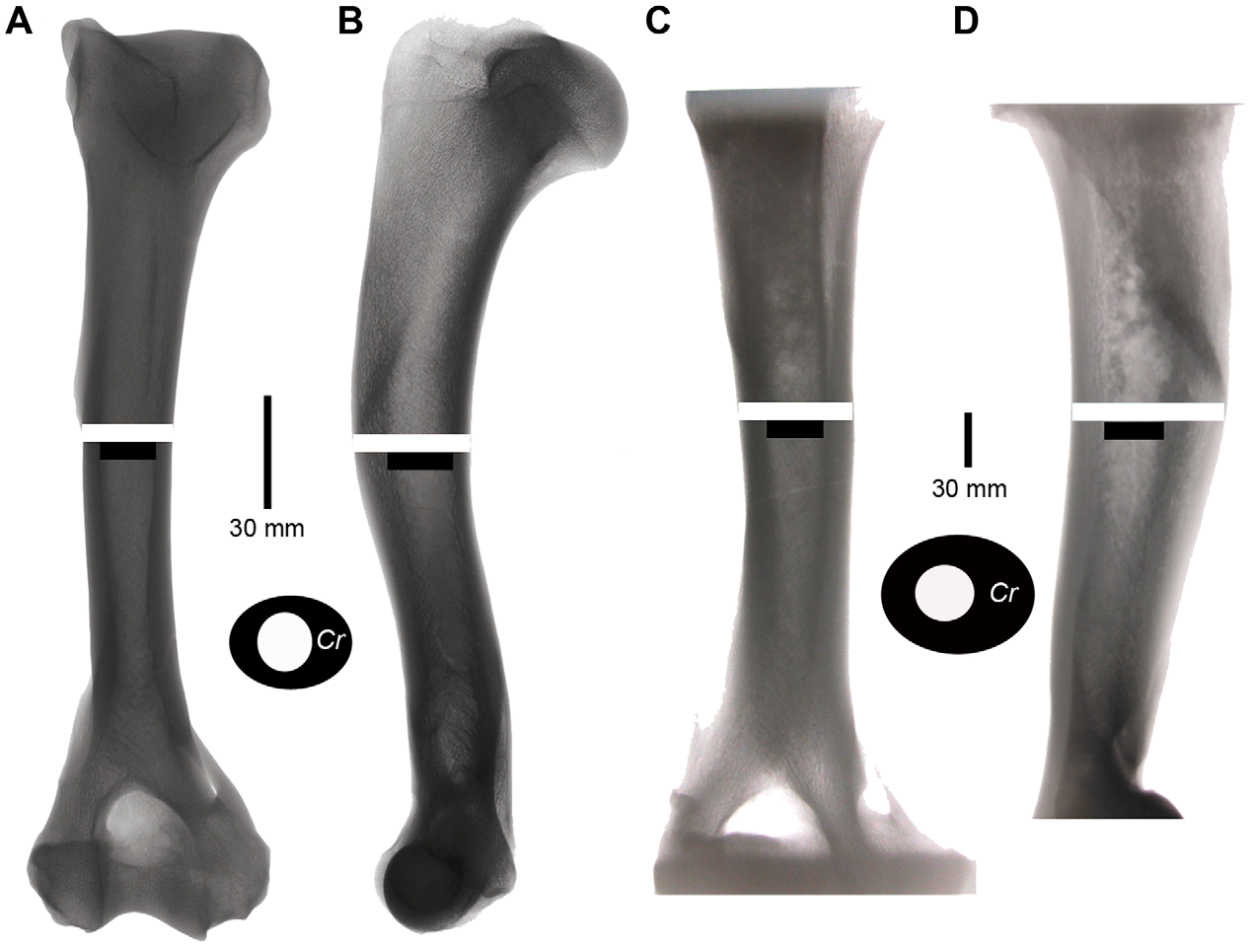
Radiographic images of jaguar, *Panthera onca*, and *Smilodon fatalis* humeri. Jaguar humerus USNM 49393 taken in the a) craniocaudal and b) mediolateral plane, and of *Smilodon fatalis* humerus LACMHC K-762 in c) craniocaudal and d) mediolateral plane. White bars indicate subperiosteal diameter and black bars indicate endosteal diameter. Between each view is a cross-sectional representation of each bone, “*Cr*” represents the cranial face of the cross-section.

When *Smilodon fatalis* was compared with all extant felids and the larger, extinct lion, *Panthera atrox*, it had humeri that were more resistant to non-axial bending (J/2) and more resistant to bending specifically in both the mediolateral and craniocaudal planes relative to bone length (Table 1, Fig. 1a–c). Although *P. atrox* is similar to *S. fatalis* with regards to bending in the craniocaudal and mediolateral planes, and average bending resistance (Ix, Iy and J/2 values respectively), its humerus is much longer. The greater rigidity of *Smilodon* humeri largely reflects a greater external diameter relative to bone length, but is also due to thicker cortical bone in *Smilodon*, suggesting that their bones were loaded more heavily in bending and axial compression than would be expected for similar-sized extant cats. The relative thickening of *Smilodon* humeri is apparent in radiographs (Fig. 2) and in comparisons of K-values (Table 2). Low K-values indicate a small marrow cavity diameter relative to external diameter. In most cats, K_ml_ is less than K_cc_ indicating the humerus is loaded more heavily in the mediolateral direction. However, *Smilodon* exhibits the lowest K_cc_ and greatest relative thickening of humeral cortical bone in the craniocaudal plane, and also ranks among the lowest values for K_ml_ as well (Table 2).

**Table 1:** Regression coefficients of log_10_ humeral or femoral cortical variables against the respective log_10_ bone (humerus or femur) length.

variable	R^2^	slope	intercept	SEE
CA humerus	0.970	2.635	−3.888	0.083
Ix humerus	0.974	5.434	−8.792	0.160
Iy humerus	0.976	5.148	−8.348	0.145
J/2 humerus	0.975	5.323	−8.630	0.153
J/2 femur	0.966	4.694	−7.526	0.169

(SEE, standard error of the estimate. *S. fatalis* was not included in any regression equations.)

**Table 2:** Mean (+/− SD) values for K craniocaudal, K mediolateral, CA, and J/2 for *Smilodon fatalis*, all other cats sampled in this study (including pantherins), and only large pantherins^1^.

	*Smilodon fatalis*		all cats		pantherins only^1^	
	humerus	femur	humerus	femur	humerus	femur
K_cc_	0.513 (0.060)	0.514 (0.059)	0.629* (0.065)	0.597* (0.061)	0.616* (0.080)	0.577* (0.046)
K_ml_	0.494 (0.081)	0.494 (0.073)	0.581* (0.082)	0.622* (0.066)	0.528* (0.082)	0.580* (0.060)
CA	3.033 (0.050)	2.805 (0.055)	2.038* (0.586)	2.101* (0.486)	2.708* (0.354)	2.593* (0.285)
J/2	5.211 (0.085)	4.743 (0.075)	3.331* (1.16)	3.447* (0.938)	4.641* (0.675)	4.393* (0.554)

1 (Pantherins include *P. atrox*, *P. leo*, *P. tigris*, *P. onca*, *P. pardus* and *P. uncia*.

(*) indicates a significant difference between *S. fatalis* and pantherins or between *S. fatalis* and all other cats in a Mann-Whitney U-test (p<0.05).)

The femur of *S. fatalis* also shows cortical thickening as evidenced by low K-values (Table 2). In both extant cats and *Smilodon*, values of K_cc_ and K_ml_ are similar for the femur. Despite the cortical thickening, the femur of *Smilodon* is similar to other cats in estimates of compressive and bending strength (Table 1, Fig. 1d).

Large values for humerus thickness in *Smilodon* were also demonstrated by CA measurements (Table 2). Both femora and humeri showed significantly higher CA when compared with all cats, or with pantherins only. However, the disparity between *Smilodon* and other groups was always greater for humeral measurements (0.995 all cats, 0.325 pantherins) than for femoral measurements (0.704 all cats, 0.212 pantherins).

All of the calculated estimates of bone strength and rigidity (CA, Ix, Iy, J/2) were positively allometric with respect to bone length in both the humerus and femur (Table 1). As also found by Doubé et al. [29], the humerus shows a stronger positive allometry than the femur, perhaps because larger cats utilize their forelimbs to kill relatively larger prey [28].

## Discussion

*Smilodon* humeri were distinct from those of non-sabertooth cats: they were thicker and more resistant to bending in both the mediolateral and craniocaudal planes. Although large felids tend to have a minor advantage over smaller felids, with slightly more resistance to bending in the proximal forelimbs [28], [29], for its size, *S. fatalis* had exceptional resistance to bending in the humerus. Sorkin [32] found similar results for external measurements of the humeri of both *S. fatalis* and *P. atrox*, with both of them having relatively robust humeri, but with *Smilodon* showing increased thickening relative to length. Although the femur also exhibits cortical thickening, it falls within the range of variation seen in extant cats, and thus follows scaling expectations.

The combination of thickened cortical bone and expanded external diameter in the humerus of *S. fatalis* suggests an unusual adaptation for both large bending and compressive loads on the forelimbs. Cortical thickening helps resist buckling due to axial compression, while higher moments of area distribute bone farther from the neutral axis, increasing resistance to bending [27], [33], [34]. This is consistent with the probable presence of relatively large and forceful forelimb flexor and extensor musculature in *S. fatalis* as evidenced by prominent muscle scars and expanded attachment areas positioned to improve mechanical advantage [2], [12], [35], [36]. Like modern big cats, *S. fatalis* used its forelimbs to both apprehend and position prey for a killing bite. However, unlike modern big cats, *Smilodon* may have had to rely more heavily on its forelimbs to hold prey because of its elongate canines. Salesa et al. [37] arrived at a similar conclusion in their recent study of an early Old World ancestor of *Smilodon*, *Promegantereon ogygia*, (age 9.7–8.7 million years ago). This early sabertooth also had robust forelimbs, intermediate in strength between less-robust conical tooth cats and later sabertooth species and the authors suggested that the greater forelimb strength co-evolved with elongated saber teeth as an adaptation to protect the sabers.

Extant large cats, when killing large prey, use a prolonged suffocating bite to the throat or nose. This crushing bite adds a third point of contact and supports the forelimbs in immobilizing prey [38]. By contrast, sabertooth cats would have killed more quickly with slashing bites to the throat [1], [39] that could not have assisted greatly or at all in holding the prey [8]. Additionally, because the elongate canines were relatively vulnerable to fracture [40], it would have been critical to minimize prey struggling and position the killing bite carefully to avoid contact with bone. This likely selected for enhanced forelimb strength in *S. fatalis*.

Cross-sectional limb bone properties have been explored in only a few orders of mammals, including primates, rodents, ungulates, and carnivores, e.g. [14], [15], [17], [19], [20], [29], [33], [41]–[44]. Among these, there are two interesting partial analogs to the pattern of much greater forelimb than hind limb strength seen in *Smilodon*. The first is in a distantly related group that also uses its forelimbs in a specialized way, fossorial caviomorph rodents. The humerus of the Highland tuco-tuco (*Ctenomys opimus*) differs from other caviomorph rodents, in having thicker cortices and a higher resistance to non-axial bending (high J/2); but its femur is similar to other species [44]. Like *S. fatalis*, the tuco-tuco has enlarged forelimb muscles and its forelimbs are loaded heavily, but for different reasons. Rather than grappling with prey, tuco-tucos use their forelimbs to excavate burrows, cutting dirt with powerful movements of their forefeet. Among caviomorphs, moderate or occasional diggers do not show such extreme adaptation. Thus, in both *C. opimus* and *S. fatalis*, greater differences in forelimb and hind limb use result in parallel differences in limb structure. A second example can be found in the bush dog: this small, rarely seen South American forest canid shows thickened cortical bone in the humerus relative to other dogs, and relative to its mass [30]. Bush dogs are excellent swimmers with partially webbed feet [45], [46]; this habit might explain the increased cortical thickness in the humerus relative to the femur.

It is unlikely that the enhanced forelimb strength of *Smilodon* represents an adaptation to either digging or swimming, rather than prey-killing, given that its distal unguals are retractile and shaped like those of felids rather than diggers [47] and a specialization for swimming would be quite surprising among felids. Another alternative explanation for enhanced forelimb strength in *Smilodon* might be as an adaptation to climbing given that skeletal adaptations of the forelimbs for climbing and prey-killing are similar in felids [28]. However, the largest extant felids (lions, tigers) and ursid (*U. arctos*) rarely climb as adults, probably because their mass makes climbing too difficult and dangerous [48]–[50].

Bones with thick cortices are heavier and are energetically more costly to build, maintain, and move. Their presence in *S. fatalis* strongly suggests a forelimb dominated predation strategy that differed from that of modern felids, and hence corroborates conclusions based on craniodental and neck anatomy [1], [6], [9], [39], [51]. The extreme specialization of the skull, teeth, neck and forelimbs of *Smilodon* probably made it an efficient predator of large ungulate prey, such as bison and camels [52], and, perhaps, juvenile proboscideans. Unfortunately, this specialization may also have led to *Smilodon*'s extinction, as the cat may have been too specialized to switch to alternative, perhaps more agile prey, such as cervids during the ice age megafaunal extinctions [53].

## Materials and Methods

Humeral and femoral cortical areas were calculated using radiographic procedures following previous studies [15], [16], with radiographs taken in both craniocaudal and mediolateral planes (Fig. 2). JMS radiographed humeri of 26 of 28 extant species at the Natural History Museum of the Smithsonian Institution (USNM) using a digital x-ray machine. The remaining two extant species humeri, all extinct species, and all femora were x-rayed by placing bones directly on a Dupont Quanta Rapid x-ray cassette containing 3M green light sensitive UVL film and using a portable x-ray machine. To equalize the effects of parallax for all specimens using the latter method, the x-ray machine was placed at a constant height above the film and external measurements were also taken directly from the bone. A measured difference of less than 4% (<3 mm) was found between the radiograph and the actual bone using this method for *Panthera atrox*, the largest species radiographed.

Cortical thicknesses and, when possible, lengths were measured from digital radiographs using ImageJ [54] and from traditional radiographs to the nearest 0.1 mm using a light box and digital calipers. Table S1 includes a list of species measured and individual radiographic measurements and calculations.

Measurements of internal and external diameters were taken for both humerus and femur approximately at the midshaft, taking humerus measurements immediately distal to the deltopectoral crest to minimize interference from this muscle insertion area. These measures were used to estimate aspects of long bone strength in axial compression (CA), bending about mediolateral and craniocaudal planes (Ix, Iy, respectively), and average rigidity in non-axial loading (J/2), [15], [16], [18], [42], [43]. Values were calculated using the following formulas:
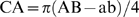


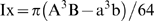


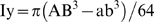


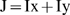
where A = external craniocaudal diameter, B = external mediolateral diameter, a = craniocaudal diameter of the medullary cavity, and b = mediolateral diameter of the medullary cavity [15], [16], [43]. One additional measure of relative cortical thickness (K) was assessed that is independent of bone length, measured in the craniocaudal (cc) and mediolateral directions (ml) as:
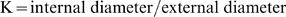
where values closer to one signify relatively thinner cortical bone and values closer to zero signify relatively thicker cortical bone [55].

To assess differences between species, species averages were calculated for CA, Ix, Iy, J/2, K_cc_, K_ml_, and lengths. All measurements except K were log_10_ transformed and regressed against respective log_10_ bone (humerus or femur) length. Differences between *Smilodon* and all other felids, and *Smilodon* and the clade that includes only large felids (pantherins) were analyzed using non-parametric Mann-Whitney U-tests.

## Supporting Information

Table S1 List of species/specimens measured; number and letter abbreviations for Figure 1; sex, specimen number, limb element, raw measurement data and calculations of CA, Ix, Iy, and J.
(0.23 MB DOC)
