# Supplementary material for: Radiographs Reveal Exceptional Forelimb Strength in the Sabertooth Cat, Smilodon fatalis
Source: PLoS One. 2010 Jul 2;5(7):e11412. doi: 10.1371/journal.pone.0011412 (PMC2896400; doi:10.1371/journal.pone.0011412)
Supplement: Table S1 — List of species/specimens measured; number and letter abbreviations for Figure 1; sex, specimen number, limb element, raw measurement data and calculations of CA, Ix, Iy, and J. (0.23 MB DOC) [file pone.0011412.s001.doc]

Table S1. List of species/specimens measured, number and letter abbreviations for Figure 1, sex, specimen number, limb element, raw measurement data and calculations of CA, Ix, Iy, and J. All measurements in mm, unless otherwise specified.

| Species | Sp. No. | sex | specimen no. | element | external ML diameter | internal ML diameter | external CC diameter | internal CC diameter | Length | CA (mm^2^) | Ix (mm^4^) | Iy (mm^4^) | J (mm^4^) |
| --- | --- | --- | --- | --- | --- | --- | --- | --- | --- | --- | --- | --- | --- |
| *Acinonyx jubatus* | 1 | male | USNM 161922 | humerus | 18.04 | 10.08 | 25.36 | 15.36 | 248.33 | 237.713 | 6536.285 | 12649.79 | 19186.08 |
| *Acinonyx jubatus* | 1 | female | USNM 162929 | humerus | 17.56 | 10.97 | 24.57 | 16.62 | 238.83 | 195.6645 | 5453.52 | 10313.15 | 15766.67 |
| *Acinonyx jubatus* | 1 | ? | LACM 72506 | femur | 21.13 | 12.57 | 21.82 | 11.4 | 293.33 | 249.567 | 8993.267 | 9861.251 | 18854.52 |
| *Acinonyx jubatus* | 1 | female | UCLA 1961 | femur | 16.92 | 11.03 | 17.9 | 11.7 | 255.12 | 136.5156 | 3485.525 | 3896.367 | 7381.892 |
| *Caracal aurata* | 2 | male | USNM 278523 | humerus | 11.77 | 7.25 | 16.54 | 11.07 | 155.49 | 89.86392 | 1116.761 | 2131.503 | 3248.264 |
| *Caracal aurata* | 2 | male | USNM 282637 | humerus | 12.04 | 8.03 | 16.06 | 10.28 | 167.95 | 87.03311 | 1114.643 | 2019.904 | 3134.547 |
| *Caracal caracal* | 3 | male | LACM 42383 | humerus | 11.96 | 9.41 | 14.52 | 11.3 | 156.5 | 52.87787 | 757.1682 | 1130.727 | 1887.895 |
| *Caracal caracal* | 3 | female | USNM 384162 | humerus | 10.4 | 6.77 | 11.45 | 6.61 | 147.83 | 58.37888 | 531.5518 | 670.3608 | 1201.913 |
| *Caracal caracal* | 3 | male | USNM 396160 | humerus | 10.64 | 7.13 | 12.09 | 7.74 | 147.09 | 57.68859 | 577.1457 | 760.6896 | 1337.835 |
| *Caracal caracal* | 3 | male | LACM 42383 | femur | 13.23 | 9.46 | 13.7 | 9.16 | 194.82 | 74.29662 | 1176.63 | 1313.003 | 2489.633 |
| *Caracal serval* | 4 | female | USNM 163092 | humerus | 10.48 | 6.93 | 11.78 | 8.57 | 159.2 | 50.31598 | 525.5709 | 626.8297 | 1152.401 |
| *Caracal serval* | 4 | male | USNM 258240 | humerus | 10.76 | 6.1 | 13.17 | 7.71 | 185.06 | 74.36008 | 719.4604 | 1069.299 | 1788.76 |
| *Caracal serval* | 4 | ? | LACM 90836 | femur | 12.09 | 8.01 | 12.31 | 8.14 | 198.17 | 65.6801 | 862.493 | 894.9897 | 1757.483 |
| *Felis chaus* | 5 | female | USNM 173375 | humerus | 8.83 | 5.92 | 9.83 | 6.29 | 129.45 | 38.92598 | 268.1447 | 339.3922 | 607.5369 |
| *Felis chaus* | 5 | female | USNM 398570 | humerus | 8.58 | 5.32 | 9.83 | 5.48 | 136.55 | 43.3444 | 264.2763 | 357.0775 | 621.3538 |
| *Felis nigripes* | 6 | male | USNM 395135 | humerus | 5.8 | 3.5 | 6.16 | 3.74 | 87.06 | 17.77984 | 51.12635 | 57.56098 | 108.6873 |
| *Felis nigripes* | 6 | female | USNM 395519 | humerus | 5.08 | 2.9 | 5.44 | 3.38 | 79.2 | 14.00616 | 30.9609 | 34.64799 | 65.60889 |
| *Felis silvestris* | 7 | female | USNM 327090 | humerus | 7.2 | 4.65 | 8.81 | 5.47 | 112.06 | 29.84238 | 134.4177 | 204.316 | 338.7337 |
| *Felis silvestris* | 7 | male | LACM 56696 | humerus | 7.52 | 4.49 | 8.44 | 5.0 | 119.7 | 32.21609 | 153.9671 | 194.3792 | 348.3463 |
| *Felis silvestris* | 7 | male | USNM 450971 | humerus | 7.56 | 4.14 | 7.98 | 5.0 | 107.89 | 31.12439 | 151.838 | 163.1793 | 315.0173 |
| *Felis silvestris* | 7 | male | LACM 56696 | femur | 8.93 | 5.78 | 8.08 | 5.26 | 135.12 | 32.79163 | 232.5876 | 189.945 | 422.5327 |
| *Leopardus colocolo* | 8 | ? | USNM 172785 | humerus | 5.68 | 3.38 | 7.25 | 4.95 | 87.41 | 19.2022 | 55.83317 | 86.12737 | 141.9605 |
| *Leopardus colocolo* | 8 | male | USNM 540876 | humerus | 6.41 | 3.26 | 7.25 | 3.71 | 111.06 | 27.00034 | 87.4212 | 111.7346 | 199.1558 |
| *Leopardus geoffroyi* | 9 | female | USNM 297847 | humerus | 6.77 | 4.23 | 8.34 | 5.56 | 101.25 | 25.87337 | 106.3718 | 157.0886 | 263.4604 |
| *Leopardus geoffroyi* | 9 | female | USNM 574136 | humerus | 6.04 | 3.62 | 6.77 | 4.03 | 95.28 | 20.6577 | 63.84241 | 80.36645 | 144.2089 |
| *Leopardus pardalis* | 10 | male | USNM 256918 | humerus | 8.87 | 5.32 | 12.14 | 8.57 | 137.76 | 48.7649 | 352.5307 | 614.6512 | 967.1819 |
| *Leopardus pardalis* | 10 | female | USNM 287774 | humerus | 9.79 | 5.56 | 12.09 | 7.31 | 136.7 | 61.03918 | 495.1824 | 742.6323 | 1237.815 |
| *Leopardus pardalis* | 10 | male | UCLA 1673 | femur | 9.58 | 5.82 | 8.51 | 4.22 | 137.63 | 44.74052 | 326.4421 | 268.3472 | 594.7894 |
| *Leopardus tigrina* | 11 | male | USNM 395089 | humerus | 5.75 | 3.14 | 6.66 | 3.57 | 99.68 | 21.27267 | 56.72559 | 76.36671 | 133.0923 |
| *Leopardus wiedii* | 12 | male | USNM 240860 | humerus | 7.43 | 4.26 | 8.56 | 5.51 | 107.45 | 31.51661 | 151.4397 | 193.7788 | 345.2185 |
| *Leopardus wiedii* | 12 | female | USNM 339683 | humerus | 6.95 | 3.41 | 7.93 | 4.94 | 104.47 | 30.05569 | 121.0613 | 149.9481 | 271.0094 |
| *Lynx canadensis* | 13 | female | UCLA 13089 | humerus | 11.24 | 7.84 | 13.71 | 9.6 | 166.9 | 61.91796 | 728.5809 | 1081.347 | 1809.927 |
| *Lynx canadensis* | 13 | male | UCLA 13098 | humerus | 12.16 | 8.31 | 14.98 | 10.08 | 175.9 | 77.27689 | 1038.211 | 1588.714 | 2626.925 |
| *Lynx canadensis* | 13 | male | UCLA 13109 | femur | 14.71 | 9.74 | 13.82 | 8.76 | 219.72 | 92.65326 | 1761.985 | 1584.532 | 3346.518 |
| *Lynx canadensis* | 13 | female | UCLA 15351 | femur | 12.87 | 8.95 | 12.78 | 7.99 | 205.34 | 73.01697 | 1056.142 | 1094.589 | 2150.732 |
| *Lynx lynx* | 14 | ? | USNM 1034 | humerus | 15.23 | 10.23 | 20.48 | 14.21 | 209.93 | 130.8019 | 2804.623 | 4980.963 | 7785.586 |
| *Lynx lynx* | 14 | female | USNM 84090 | humerus | 12.21 | 6.74 | 15.26 | 8.99 | 175.48 | 98.74968 | 1228.434 | 1889.465 | 3117.899 |
| *Lynx pardinus* | 15 | male | USNM 152619 | humerus | 10.64 | 5.48 | 14.16 | 8.81 | 154.05 | 80.41189 | 766.0871 | 1298.923 | 2065.01 |
| *Lynx rufus* | 16 | male | UCLA 13933 | humerus | 9.79 | 5.49 | 11.23 | 6.86 | 144.05 | 56.76881 | 461.5267 | 593.6008 | 1055.127 |
| *Lynx rufus* | 16 | female | UCLA 13943 | humerus | 9.92 | 6.09 | 10.98 | 6.67 | 134.58 | 53.64371 | 452.1956 | 555.8891 | 1008.085 |
| *Lynx rufus* | 16 | female | LACM 52222 | femur | 9.35 | 5.82 | 8.64 | 5.12 | 154.99 | 40.044 | 297.1257 | 257.6767 | 554.8024 |
| *Lynx rufus* | 16 | female | LACM 52220 | femur | 11.13 | 7.41 | 9.89 | 6.43 | 152.9 | 49.03194 | 540.9267 | 431.8121 | 972.7388 |
| *Lynx rufus* | 16 | male | LACM 52214 | femur | 12.02 | 8.04 | 10.62 | 6.7 | 170.3 | 57.95013 | 734.404 | 588.021 | 1322.425 |
| *Lynx rufus* | 16 | male | LACM 45027 | femur | 11.1 | 7.11 | 10.64 | 6.67 | 172.55 | 55.51218 | 596.6192 | 552.7572 | 1149.376 |
| *Neofelis nebulosa* | 17 | male | USNM 49974 | humerus | 14.13 | 6.74 | 18.31 | 10.12 | 175.04 | 149.6274 | 2383.523 | 3914.817 | 6298.34 |
| *Neofelis nebulosa* | 17 | female | USNM 198705 | humerus | 10.16 | 5.64 | 12.5 | 7.85 | 137.05 | 64.97285 | 574.3868 | 840.1539 | 1414.541 |
| *Otocolobus manul* | 18 | female | LACM 90728 | femur | 8.21 | 5.28 | 6.53 | 4.09 | 102.1 | 25.14539 | 147.8307 | 94.48278 | 242.3135 |
| *Panthera leo* | 19 | female | USNM 161914 | humerus | 26.22 | 13.65 | 33.53 | 17.43 | 307.3 | 503.6264 | 27492.87 | 44969.95 | 72462.82 |
| *Panthera leo* | 19 | male | USNM 162919 | humerus | 28.43 | 13.01 | 42.85 | 26.66 | 358.11 | 684.3794 | 45452.05 | 97698.04 | 143150.1 |
| *Panthera leo* | 19 | female | LACM 171 | femur | 31.2 | 18.97 | 27.97 | 16.97 | 345.76 | 432.5526 | 36012.43 | 28961.37 | 64973.81 |
| *Panthera leo* | 19 | ? | UCLA 2470 | femur | 33.27 | 19.76 | 31.19 | 17.1 | 337.16 | 549.6179 | 49906.12 | 44702.83 | 94608.95 |
| *Panthera onca* | 20 | ? | USNM 12296 | humerus | 20.24 | 12.28 | 27.52 | 18.11 | 236.59 | 262.8052 | 9554.618 | 17127.07 | 26681.69 |
| *Panthera onca* | 20 | ? | USNM 49393 | humerus | 24.81 | 14.94 | 30.58 | 19.52 | 256.87 | 366.8288 | 19728.63 | 29371.88 | 49100.51 |
| *Panthera onca* | 20 | ? | UCLA 183 | femur | 21.63 | 14.34 | 22.01 | 13.43 | 273.47 | 222.6526 | 8989.52 | 9615.956 | 18605.48 |
| *Panthera pardus* | 21 | male | LACM 31068 | humerus | 16.78 | 11.82 | 23.52 | 19.3 | 218.9 | 130.7999 | 3890.339 | 6545.831 | 10436.17 |
| *Panthera pardus* | 21 | female | USNM 161911 | humerus | 16.7 | 8.03 | 19.39 | 11.68 | 196.91 | 180.6593 | 4136.128 | 5348.048 | 9484.176 |
| *Panthera pardus* | 21 | male | USNM 164763 | humerus | 19.51 | 11.7 | 24.63 | 16.09 | 254.73 | 229.5548 | 7713.568 | 11917.02 | 19630.59 |
| *Panthera pardus* | 21 | ? | LACM 648 | humerus | 16.79 | 10.91 | 23.72 | 14.99 | 210.1 | 184.347 | 4555.546 | 9195.443 | 13750.99 |
| *Panthera pardus* | 21 | male | LACM 31068 | femur | 20.76 | 13.1 | 18.08 | 10.73 | 251.49 | 184.394 | 6756.459 | 5228.316 | 11984.78 |
| *Panthera pardus* | 21 | ? | LACM 648 | femur | 19.37 | 12.49 | 18.46 | 11.2 | 252.16 | 170.9672 | 5514.314 | 5119.923 | 10634.24 |
| *Panthera tigris* | 22 | ? | USNM 49728 | humerus | 32.78 | 20.07 | 38.55 | 24.73 | 325.81 | 602.6658 | 56839.44 | 77283.13 | 134122.6 |
| *Panthera tigris* | 22 | female | USNM 174981 | humerus | 23.58 | 12.04 | 29.33 | 16.94 | 290.87 | 382.9945 | 17424.83 | 26331.5 | 43756.33 |
| *Panthera tigris* | 22 | ? | LACM 54239 | femur | 28.49 | 14.4 | 29.34 | 16.94 | 355.33 | 464.9248 | 30821.87 | 31885.63 | 62707.51 |
| *Panthera tigris* | 22 | ? | LACM 51573 | femur | 27.36 | 15.06 | 25.67 | 12.57 | 354.06 | 402.9305 | 23699.83 | 21249.41 | 44949.23 |
| *Panthera uncia* | 23 | female | USNM 84091 | humerus | 13.81 | 6.9 | 19.51 | 11.08 | 193.41 | 151.567 | 2343.694 | 4573.536 | 6917.23 |
| *Panthera uncia* | 23 | female | USNM 321948 | humerus | 16.34 | 9.51 | 20.48 | 12.04 | 210.44 | 172.8997 | 3877.552 | 6075.122 | 9952.674 |
| *Panthera uncia* | 23 | ? | LACM 90781 | femur | 19.7 | 11.27 | 18.16 | 9.58 | 249.46 | 196.181 | 6142.148 | 5305.005 | 11447.15 |
| *Panthera uncia* | 23 | male | LACM 90729 | femur | 19.61 | 11.92 | 17.12 | 9.49 | 243.41 | 174.8315 | 5548.354 | 4330.043 | 9878.397 |
| *Pardofelis marmorata* | 24 | male | USNM 239326 | humerus | 7.56 | 4.26 | 8.73 | 5.91 | 102.39 | 32.06168 | 162.7332 | 203.7416 | 366.4748 |
| *Pardofelis marmorata* | 24 | female | LACM 74126 | femur | 8.54 | 4.76 | 7.6 | 3.56 | 123.84 | 37.66644 | 213.5108 | 173.4794 | 386.9902 |
| *Pardofelis marmorata* | 24 | female | LACM 62826 | femur | 8.36 | 5.11 |  |  | 119.7 | 0 | 0 | 0 | 0 |
| *Pardofelis marmorata* | 24 | male | LACM 90779 | femur | 9.65 | 6.75 | 9.7 | 5.65 | 143.78 | 43.56407 | 342.5854 | 372.5659 | 715.1512 |
| *Pardofelis temminckii* | 25 | ? | USNM 258552 | humerus | 7.73 | 5.11 | 9.88 | 6.66 | 121.93 | 33.2536 | 180.3868 | 291.8495 | 472.2363 |
| *Pardofelis temminckii* | 25 | female | USNM 362188 | humerus | 10.16 | 5.96 | 12.61 | 8.45 | 141.24 | 61.0691 | 561.3671 | 823.5035 | 1384.871 |
| *Prionailurus bengalensis* | 26 | female | USNM 201069 | humerus | 6.65 | 4.23 | 7.61 | 4.71 | 100.75 | 24.09853 | 92.35597 | 122.1661 | 214.5221 |
| *Prionailurus bengalensis* | 26 | male | USNM 240006 | humerus | 7.67 | 4.12 | 9.88 | 6.07 | 120.16 | 39.87561 | 197.9953 | 317.8775 | 515.8729 |
| *Prionailurus bengalensis* | 26 | ? | LACM 90780 | femur | 7.37 | 3.72 | 6.57 | 3.46 | 117.82 | 27.92067 | 120.3601 | 95.0329 | 215.393 |
| *Prionailurus bengalensis* | 26 | male | LACM 90837 | femur | 9.52 | 7.01 | 7.74 | 5.32 | 116.66 | 28.5819 | 237.8525 | 164.8743 | 402.7268 |
| *Prionailurus planiceps* | 27 | female | USNM 145593 | humerus | 5.73 | 2.8 | 7.29 | 4.54 | 83.21 | 22.82343 | 62.43051 | 96.10842 | 158.5389 |
| *Prionailurus planiceps* | 27 | male | USNM 49973 | humerus | 5.12 | 2.56 | 6.26 | 3.85 | 82.29 | 17.43207 | 38.0727 | 54.48302 | 92.55572 |
| *Prionailurus viverrinus* | 28 | female | LACM 90838 | femur | 11.34 | 7.35 | 9.48 | 5.55 | 138.77 | 52.39446 | 570.431 | 412.5718 | 983.0029 |
| *Puma concolor* | 29 | ? | LACM 54511 | humerus | 20.39 | 15.1 | 28.9 | 19.43 | 236.6 | 232.382 | 8742.174 | 18722.02 | 27464.19 |
| *Puma concolor* | 29 | ? | LACM 70296 | humerus | 17.33 | 12.14 | 21.57 | 16.6 | 208.5 | 135.3116 | 4052.892 | 5811.349 | 9864.242 |
| *Puma concolor* | 29 | female | LACM 85438 | humerus | 16.31 | 11.05 | 22.64 | 16.01 | 198.9 | 151.0697 | 3761.442 | 7064.898 | 10826.34 |
| *Puma concolor* | 29 | male | LACM 87430 | femur | 21.74 | 12.89 | 23.05 | 12.84 | 269.04 | 263.5791 | 10275.83 | 11729.57 | 22005.41 |
| *Puma concolor* | 29 | female | LACM 85438 | femur | 17.89 | 10.63 | 17.94 | 9.92 | 248.42 | 169.2509 | 4457.338 | 4561.087 | 9018.425 |
| *Puma concolor* | 29 | female | LACM 70296 | femur | 19.11 | 11.73 | 18.42 | 10.86 | 256.76 | 176.4149 | 5449.791 | 5125.232 | 10575.02 |
| *Puma concolor* | 29 | male | LACM 54511 | femur | 21.36 | 14.18 | 22.46 | 14.35 | 284.36 | 216.9761 | 8736.029 | 9822.715 | 18558.74 |
| *Puma yagouroundi* | 30 | male | USNM 49716 | humerus | 8.25 | 5.58 | 10.23 | 7.14 | 111.58 | 34.99444 | 221.0794 | 333.8607 | 554.9401 |
| *Puma yagouroundi* | 30 | female | USNM 153531 | humerus | 7.9 | 5.34 | 8.92 | 6.19 | 104.8 | 29.38442 | 169.6134 | 213.057 | 382.6704 |
| *Puma yagouroundi* | 30 | female | LACM 30867 | femur | 9.12 | 7.04 | 8.58 | 6.54 | 106.52 | 25.2961 | 207.4663 | 186.0992 | 393.5654 |
| *Smilodon fatalis*† | SFA |  | LACMHC 3-923 | humerus | 39.2 | 16.52 | 51.76 | 30.2 | 341.42 | 1201.728 | 146362.6 | 244496.8 | 390859.3 |
| *Smilodon fatalis*† | SFA |  | LACMHC 3-946 | humerus | 34.34 | 15.9 | 47.45 | 22.2 | 316.63 | 1002.524 | 89940.29 | 171546.2 | 261486.5 |
| *Smilodon fatalis*† | SFA |  | LACMHC 61-983 | humerus | 31.16 | 19.27 | 47.3 | 21.32 | 315.86 | 834.9031 | 62757.66 | 152697.5 | 215455.1 |
| *Smilodon fatalis*† | SFA |  | LACMHC 61-762 | humerus | 37.33 | 16.31 | 54.71 | 31.43 | 326.43 | 1201.424 | 133010.7 | 275216.3 | 408227 |
| *Smilodon fatalis*† | SFA |  | LACMHC 13-525 | humerus | 40.64 | 20.47 | 50.02 | 24.04 | 333.45 | 1210.074 | 154684.8 | 235703.1 | 390387.9 |
| *Smilodon fatalis*† | SFA |  | LACMHC 13-532 | humerus | 36.63 | 17.7 | 50.59 | 30.51 | 348 | 1031.294 | 113747.3 | 208133.9 | 321881.1 |
| *Smilodon fatalis*† | SFA |  | LACMHC 67-485 | humerus | 37.83 | 20.53 | 46.68 | 19.47 | 342.12 | 1072.999 | 115783.8 | 181447.6 | 297231.3 |
| *Smilodon fatalis*† | SFA |  | LACMHC 67-474 | humerus | 38.55 | 18.5 | 49.59 | 26.27 | 353.16 | 1119.742 | 131291.2 | 214305.1 | 345596.2 |
| *Smilodon fatalis*† | SFA |  | LACMHC 77-450 | humerus | 36.95 | 13.67 | 49.89 | 25.81 | 330.76 | 1170.725 | 120309 | 213692 | 334001.1 |
| *Smilodon fatalis*† | SFA |  | LACMHC 77-449 | humerus | 37.41 | 23.36 | 51.61 | 26.54 | 323.09 | 1029.465 | 116030.7 | 231004.5 | 347035.2 |
| *Smilodon fatalis*† | SFA |  | LACMHC K3695 | femur | 32.93 | 17.58 | 31.33 | 18.81 | 368.3 | 550.5775 | 49900.16 | 43966.67 | 93866.84 |
| *Smilodon fatalis*† | SFA |  | LACMHC K3694 | femur | 36.65 | 21.01 | 32.69 | 18.64 | 378.46 | 633.3938 | 70510.48 | 56168.25 | 126678.7 |
| *Smilodon fatalis*† | SFA |  | LACMHC K3776 | femur | 31.54 | 17.99 | 30.35 | 16.05 | 375.92 | 525.0383 | 42155.58 | 39630.91 | 81786.5 |
| *Smilodon fatalis*† | SFA |  | LACMHC K3230 | femur | 37.89 | 17.8 | 32.79 | 14.55 | 379.73 | 772.3787 | 83527.81 | 62880.64 | 146408.5 |
| *Smilodon fatalis*† | SFA |  | LACMHC K3220 | femur | 37.34 | 15.92 | 31.74 | 15.94 | 375.92 | 731.5252 | 77957.64 | 55444.06 | 133401.7 |
| *Smilodon fatalis*† | SFA |  | LACMHC K3236 | femur | 31.56 | 11.11 | 34.23 | 14.71 | 355.6 | 720.1086 | 51828.5 | 60397.86 | 112226.4 |
| *Smilodon fatalis*† | SFA |  | LACMHC 43223 | femur | 37.25 | 18.29 | 30.72 | 14.06 | 382.27 | 696.7759 | 73718.95 | 50514.85 | 124233.8 |
| *Smilodon fatalis*† | SFA |  | LACMHC K3164 | femur | 33.85 | 15.38 | 32.53 | 15.23 | 381 | 680.8641 | 59214.31 | 54530.97 | 113745.3 |
| *Smilodon fatalis*† | SFA |  | LACMHC K3193 | femur | 34.61 | 14.97 | 35.47 | 16.11 | 358.14 | 774.756 | 69530.2 | 72742.58 | 142272.8 |
| *Smilodon fatalis*† | SFA |  | LACMHC K3486 | femur | 33.91 | 17.96 | 32.5 | 15.62 | 358.14 | 645.2358 | 57764.71 | 53781.14 | 111545.9 |
| *Smilodon fatalis*† | SFA |  | LACMHC K3473 | femur | 34.46 | 16.2 | 30.83 | 16.01 | 356.87 | 630.706 | 58587.08 | 46305.18 | 104892.3 |
| *Smilodon fatalis*† | SFA |  | LACMHC K3475 | femur | 32.53 | 13.11 | 29.2 | 15.06 | 335.28 | 590.9645 | 47674.93 | 37557.88 | 85232.82 |
| *Smilodon fatalis*† | SFA |  | LACMHC K3281 | femur | 34.54 | 20.58 | 30.87 | 17.55 | 353.06 | 553.7613 | 54932.51 | 44416.53 | 99349.04 |
| *Smilodon fatalis*† | SFA |  | LACMHC K3285 | femur | 35.75 | 21.45 | 32.45 | 20.19 | 375.92 | 570.9939 | 62999 | 51298.17 | 114297.2 |
| *Smilodon fatalis*† | SFA |  | LACMHC K3289 | femur | 33.85 | 17.38 | 30.6 | 17.21 | 358.14 | 578.603 | 53824.51 | 43260.69 | 97085.2 |
| *Panthera atrox*† | PAT |  | LACMHC 6891 | humerus | 44.07 | 21.08 | 72.94 | 49.91 | 416.56 | 1698.316 | 283504.4 | 710831.7 | 994336 |
| *Panthera atrox*† | PAT |  | LACMHC 6890 | humerus | 35.85 | 19.07 | 62.13 | 43.63 | 411.48 | 1095.895 | 125667.4 | 344303.6 | 469971 |
| *Panthera atrox*† | PAT |  | LACMHC 6874 | humerus | 35.91 | 22.62 | 50.89 | 37.61 | 355.6 | 767.1154 | 94310.04 | 173247.1 | 267557.2 |
| *Panthera atrox*† | PAT |  | LACMHC 6904 | humerus | 30.07 | 16.78 | 52.72 | 27.58 | 356.04 | 881.6079 | 63966.73 | 199006.5 | 262973.3 |
| *Panthera atrox*† | PAT |  | LACMHC 6896 | humerus | 38.18 | 16.04 | 53.4 | 28.38 | 376.13 | 1243.754 | 140138.7 | 267386.5 | 407525.2 |
| *Panthera atrox*† | PAT |  | LACMHC 2903-R-8 | humerus | 34.91 | 13.81 | 49.17 | 29.94 | 342.93 | 1023.416 | 98817.17 | 185520.1 | 284337.3 |
| *Panthera atrox*† | PAT |  | LACMHC 6878 | humerus | 40.63 | 21.49 | 55.54 | 36.47 | 386.28 | 1156.774 | 165091.9 | 290521.8 | 455613.8 |
| *Panthera atrox*† | PAT |  | LACMHC 1557 | humerus | 33.95 | 15.7 | 47.01 | 24.73 | 350.03 | 948.5477 | 85600.5 | 161477.4 | 247077.9 |
| *Panthera atrox*† | PAT |  | LACMHC 6911 | humerus | 35.21 | 16.37 | 50.74 | 25.81 | 352.67 | 1071.319 | 103164.4 | 211964.9 | 315129.4 |
| *Panthera atrox*† | PAT |  | LACMHC 6881 | humerus | 34.11 | 14.29 | 48.63 | 26.87 | 350.75 | 1001.223 | 90888.05 | 178950.9 | 269839 |
| *Panthera atrox*† | PAT |  | LACMHC 6903 | humerus | 35.74 | 16 | 58.77 | 29.54 | 371.57 | 1278.471 | 125761.7 | 335870.9 | 461632.5 |
| *Panthera atrox*† | PAT |  | LACMHC 7143 | femur | 42.05 | 19.04 | 39.28 | 21.62 | 436.88 | 973.9559 | 136038.4 | 115652.9 | 251691.4 |
| *Panthera atrox*† | PAT |  | LACMHC 7144 | femur | 42.08 | 25.05 | 40.49 | 27.17 | 436.8 | 803.6278 | 127132.1 | 112453.2 | 239585.3 |
| *Panthera atrox*† | PAT |  | LACMHC 7192 | femur | 41.1 | 21.03 | 37.79 | 20.91 | 431.8 | 874.4872 | 119240.6 | 99440.75 | 218681.4 |
| *Panthera atrox*† | PAT |  | LACMHC 7190 | femur | 39.72 | 24.07 | 36.27 | 22.47 | 426.72 | 706.6946 | 96187.83 | 79625.1 | 175812.9 |

† represents extinct species. LACM = Natural History Museum of Los Angeles County; LACMHC = Los Angeles Museum Hancock Collection (Page Museum); UCLA = UCLA Donald R. Dickey Collection; USNM = U.S. National Museum of Natural History, Smithsonian Institution.
